# “Outside-to-inside,” “inside-to-outside,” and “intrinsic” endogenous pathogenic mechanisms in atopic dermatitis: keratinocytes as the key functional cells involved in both permeability barrier dysfunction and immunological alterations

**DOI:** 10.3389/fimmu.2023.1239251

**Published:** 2023-08-11

**Authors:** Yutaka Hatano, Peter M. Elias

**Affiliations:** ^1^Department of Dermatology, Faculty of Medicine, Oita University, Oita, Japan; ^2^Department of Dermatology, University of California, San Francisco and Veterans Affairs Health Care System, San Francisco, CA, United States

**Keywords:** atopic dermatitis, keratinocyte, permeability barrier dysfunction, allergic inflammation, filaggrin, keratin 1, PPAR alpha

## Abstract

Permeability barrier disruption has been shown to induce immunological alterations (i.e., an “outside-to-inside” pathogenic mechanism). Conversely, several inflammatory and immunological mechanisms reportedly interrupt permeability barrier homeostasis (i.e., an “inside-to-outside” pathogenic mechanism). It is now widely recognized that alterations of even a single molecule in keratinocytes can lead to not only permeability barrier dysfunction but also to immunological alterations. Such a simultaneous, bidirectional functional change by keratinocytes is herein named an “intrinsic” pathogenic mechanism. Molecules and/or pathways involved in this mechanism could be important not only as factors in disease pathogenesis but also as potential therapeutic targets for inflammatory cutaneous diseases, such as atopic dermatitis, psoriasis, and prurigo nodularis. Elevation of skin surface pH following permeability barrier abrogation comprises one of the key pathogenic phenomena of the “outside-to-inside” mechanism. Not only type 2 cytokines (e.g., IL-4, IL-13, IL-31) but also type 1 (e.g. IFN-γ), and type 3 (e.g., IL-17, IL-22) as well as several other inflammatory factors (e.g. histamine) can disrupt permeability barrier homeostasis and are all considered part of the “inside-to-outside” mechanism. Finally, examples of molecules relevant to the “intrinsic” pathogenic mechanism include keratin 1, filaggrin, and peroxisome proliferator-activated receptor-α (PPARα).

## Introduction

**Figure 1:**
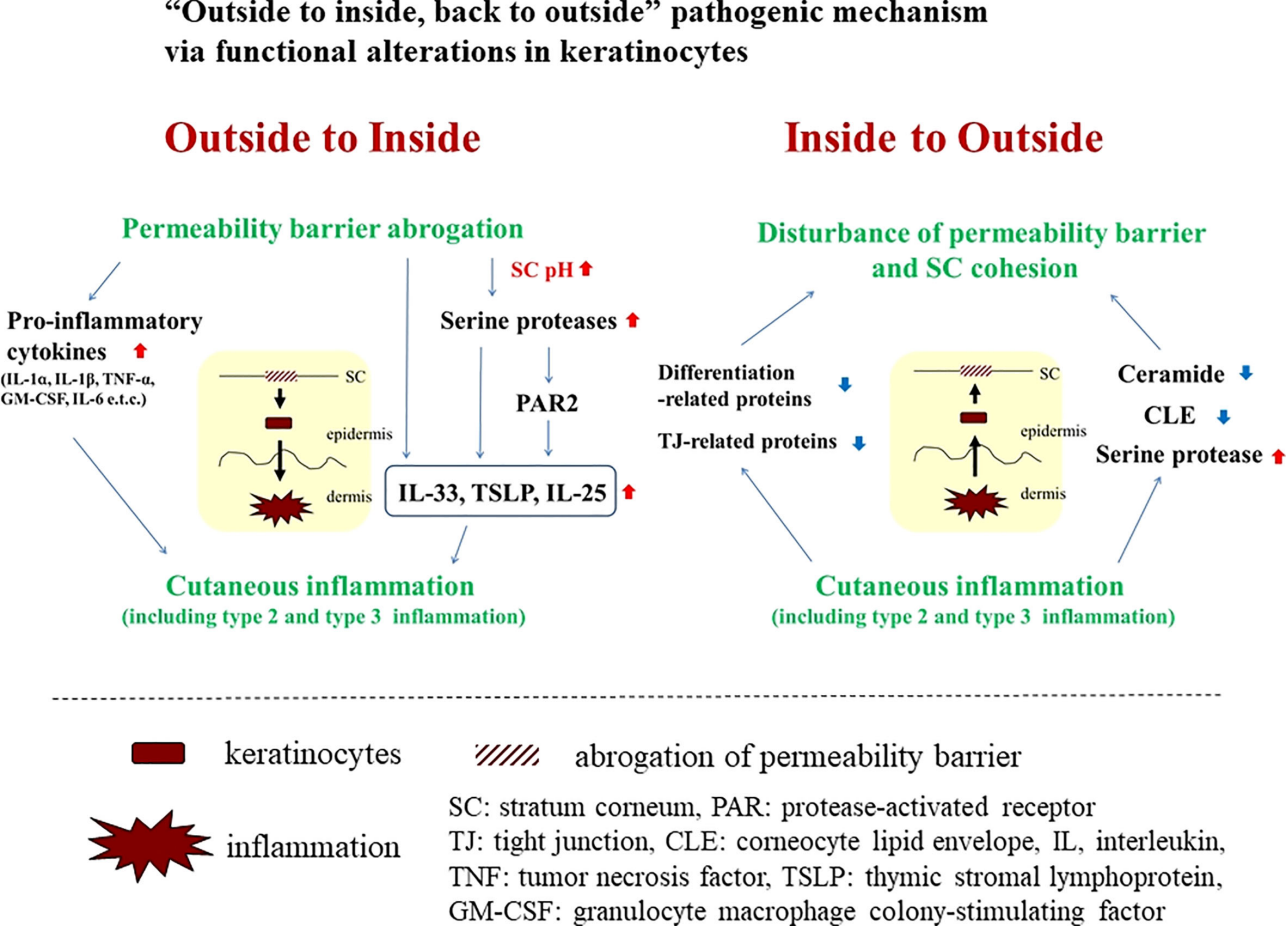
“Outside to inside, back to outside” pathogenic mechanism via functional alterations in keratinocytes.

As previously described in numerous review articles, permeability barrier abrogation has been shown to induce immunological alterations (i.e., “outside-to-inside” pathogenic mechanism; **Figure 1**). Conversely, several inflammatory and immunological factors have been reported to disturb permeability barrier homeostasis (i.e., “inside-to-outside” pathogenic mechanism; **Figure 1**) (1). In this article, we will highlight additional associations between permeability barrier abrogation and inflammatory and/or immunological alterations.

**Figure 2:**
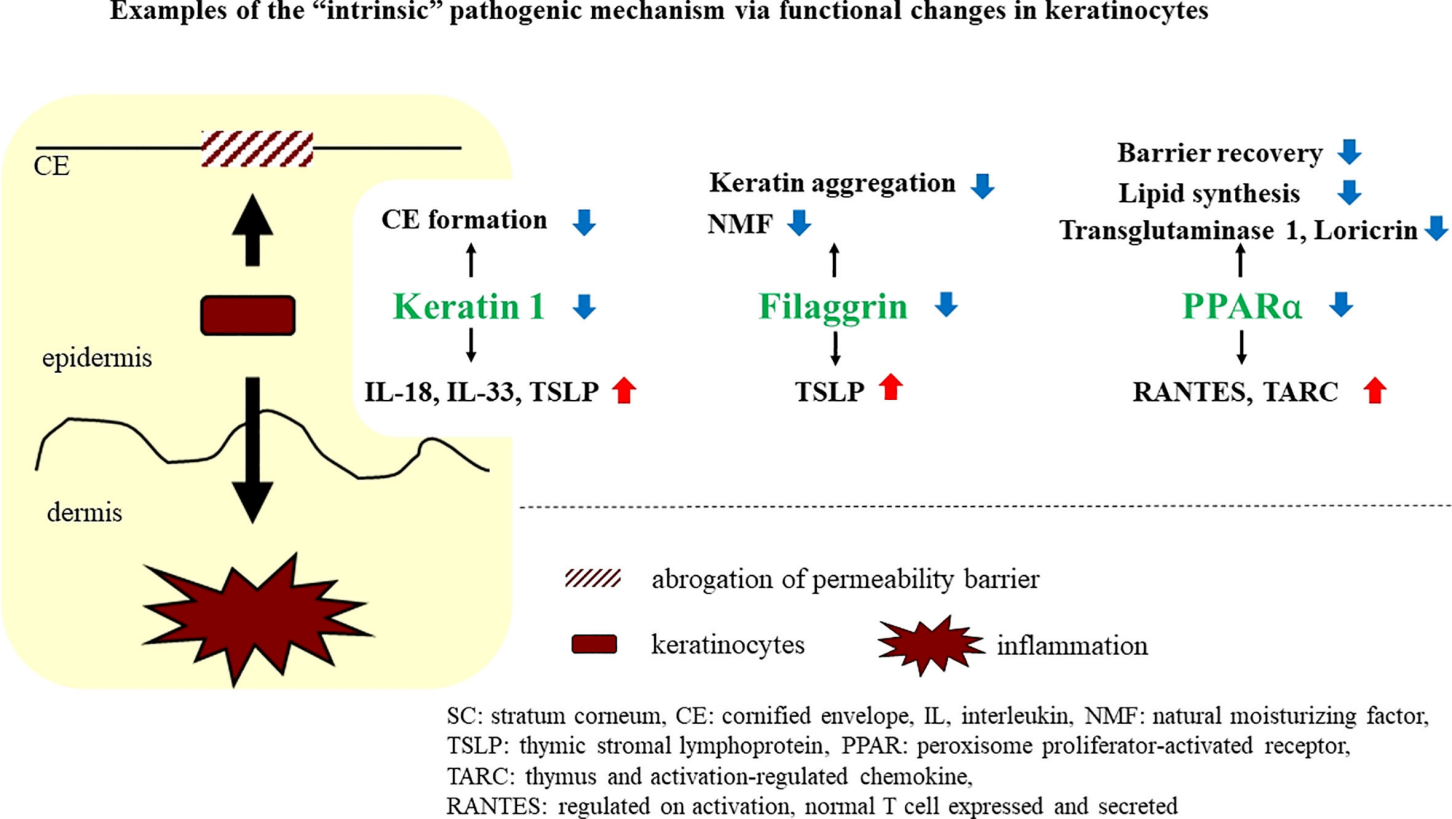
Examples of the “intrinsic” pathogenic mechanism via functional changes in keratinocytes.

Currently, multiple studies have revealed that modification in even a single molecule in keratinocytes can induce epidermal functional changes that not only disrupt permeability barrier homeostasis but also lead to immunological alterations. Here, we describe simultaneous functional changes in keratinocytes in two different directions as an “intrinsic” pathogenic mechanism (**Figure 2**).

## Induction of both type 2 and type 3 inflammation by the “outside-to-inside” pathogenic mechanism

Following epidermal permeability barrier abrogation, the “outside-to-inside” mechanism not only encompasses the induction and/or secretion of pro-inflammatory cytokines such as interleukin (IL)-1α, IL-1β, tumor necrosis factor (TNF)-α, and granulocyte macrophage colony-stimulating factor (GM-CSF) from epidermal keratinocytes (2), but also elevation of skin surface pH, accompanied by elevated kallikrein (KLK) activity (1), all key pathogenic phenomena for inducing inflammation, especially type 2. Initiation of production and/or secretion of so-called danger signals or alarmins, such as IL-25, IL-33, and thymic stromal lymphopoietin (TSLP), lead to immunological alterations of type 2 inflammation with or without protease-activated receptor (PAR)2-dependent responses (3). In addition, permeability barrier abrogation reportedly induces type 2 and type 3 inflammation (e.g., IL-17 and IL-22) via activation of KLK5 and the PAR2 axis (4). In line with this theory, combined treatment with a PAR2 inhibitor and a lactobionic acid (LBA) (‘super acid’) exhibited therapeutic effects on hapten-induced atopic dermatitis (AD)-like dermatitis in a murine model in which elevations of epidermal TSLP paralleled development of the dermatitis (5). Application of LBA during the induction phase was also reported to prevent the initial emergence of hapten-induced AD-like dermatitis (6). Notably, both type 2 and type 3 inflammation are reported to be involved in the pathogenesis of a hapten-induced AD-like dermatitis (7), supporting the concept that a combination of permeability barrier abrogation, elevation of stratum corneum pH, and PAR2 activation could lead to the induction of type 2 and type 3 inflammation.

## Several pro-inflammatory factors are involved in the “inside to outside” pathogenic mechanism

Since we first described the “inside to outside” pathogenic mechanism (1), several inflammatory factors and pathways have been found to be involved in this mechanism.

Prototypic type 2 cytokines, IL-4 and IL-13, downregulate the expression of filaggrin (FLG), loricrin (LOR), and involucrin (IVL) (8, 9). IL-4 also reduces ceramide synthesis and compromises stratum corneum (SC) cohesion (10–12). Cutaneous permeability barrier recovery is suppressed by IL-4 (13). Recently, type 2 cytokines were reported to provoke shortening of ceramide carbon chains via down-regulation of elongase expression, such as elongation of very long chain fatty acids protein (ELOVL)3 (14). Type 2 cytokines also downregulate enzymes involved in cornified lipid envelope (CLE) formation, such as ALOX12B, ALOXE3, and ABHD9 (15). Likewise, IL-31 reportedly suppresses expression of FLG (16). Claudin-1, a tight junction-related protein, is downregulated by IL-4, IL-13, and IL-31 (17).

Considerable data demonstrating the harmful effects of a variety of inflammatory factors (besides type 2 cytokines) on the permeability barrier have been accumulated. Type 3 cytokines (e.g., IL-17 and IL-22) and histamines downregulate expression of FLG, LOR, and IVL (18, 19), R. Histamine reportedly downregulates the expression of claudin-1, claudin-4, occludin, and zonula occludens (ZO)-1 (19). Interferon (IFN)-γ, a prototypic type 1 cytokine, has been demonstrated to reduce expression of FLG, claudin-1, and ELOVL1 (20–22).

## Recognition of a newly emerging concept, an “intrinsic” pathogenic mechanism, elucidating the association between permeability barrier dysfunction and inflammatory reactions

Abundant evidence leads us to recognition of the association between permeability barrier dysfunction and inflammatory reactions. Alteration of even one molecule in epidermal keratinocytes can induce functional changes, leading to simultaneous permeability barrier dysfunction and inflammatory and/or immunological dysregulation. Here, such a molecule is called an “intrinsic” participant, and the pathogenic mechanism induced by the alteration of these molecules is also called “intrinsic.” Examples of “intrinsic” molecules are described below (**Figure 2**).

## Keratin 1

Keratins constitute the intermediate filament cytoskeleton in keratinocytes and play an important role in the mechanical integrity of corneocytes, during linkage to cornified envelopes, which is a critical process for competent permeability barrier formation (23, 24). In fact, permeability barrier abnormalities have been demonstrated in keratin (KRT)-deficient conditions (25, 26). Meanwhile, KRT1 abnormalities reportedly lead to cutaneous inflammation, accompanied by elevations of IL-18, IL-33, and TSLP (27), which are well-known danger signals in type 2 inflammation induction, a hallmark in AD pathogenesis (3, 28–30). Interestingly, IL-18 secretion is induced in *KRT1* knockout-cultured keratinocytes in a caspase-1-dependent manner, suggesting that the secretion of IL-18 in *KRT1*-deficient mice could be a primary effect of *KRT1* depletion, rather than a secondary effect, following permeability barrier abrogation (27). Together, downregulation of KRT1 could cause functional, dual directional alterations in keratinocytes, leading to both permeability barrier abrogation and allergic inflammation in AD. Accordingly, expression of KRT1 reportedly is downregulated in atopic lesions due to elevated levels of inflammatory cytokines, such as IL-33, IL-4, and IL-13 (31, 32). Thus, the downregulation of KRT1 could augment not only permeability barrier abrogation but also allergic inflammation.

## Filaggrin

FLG is an epidermal differentiation-related molecule, which plays important roles in both permeability barrier homeostasis and SC hydration (33, 34). In fact, epidermis in which the filaggrin gene is knocked down exhibits substantial alterations in permeability barrier function (35). Interestingly, it has been recently shown that keratinocytes transfected with siRNA against the profilaggrin gene are able to produce greater quantities (versus control) of TSLP, which is one of the essential cytokines in the induction of type 2 immunological reactions (36). Knockdown of *FLG* reportedly increases the production of interleukin (IL)-1α, IL-8, IL-18 and GM-CSF in stratified human keratinocytes (37). It has also been reported that keratinocytes of flaky tail (versus wild-type mice), in which FLG is deficient due to a loss-of-function mutation in *profilaggrin*, produce more of the proinflammatory cytokine, IL-1β (38). In ichthyosis vulgaris, which is caused by loss of function mutations in *FLG*, expression of pro-inflammatory cytokines increases. Together, these results show that an abnormality in FLG, which has been mainly regarded as a barrier-related molecule, could simultaneously modulate processes leading to allergic inflammation in skin (39). Whether such a functional change in keratinocytes towards a proinflammatory phenotype is attributable to dysfunction of FLG in KRT aggregation remains undetermined, although in cultured epidermal keratinocytes knocked down of *FLG*, KRT1 expression was reported to be unaffected (40).

## Peroxisome proliferators-activated receptor α

Peroxisome proliferators-activated receptors (PPARs) belong to the nuclear hormone receptor class II and have three subtypes, PPARα, PPARβ/δ and PPARγ (41). They are called liposensors because their ligands are lipids or lipid derivatives. Generally, PPAR signaling has positive effects on barrier homeostasis, but it can also have anti-inflammatory effects, although there are some differences in the impact of their subtypes (41). The activation of PPARs stimulates lipid synthesis and epidermal differentiation, while also accelerating recovery after permeability barrier disruption (41). Moreover, epidermal barrier development is delayed in PPARα-deficient mice (42).

Activators of PPARα suppress both allergic and irritant cutaneous inflammation *in vivo* (43). Interestingly, it has been reported that PPARα expression in the skin is reduced in patients with AD and that PPARα-deficient mice develop more severe hapten-induced AD-like dermatitis than wild-type mice (44). RNA sequence analysis also revealed that PPARα expression is down-regulated in AD-like lesions compared with those in non-lesional flaky tail mice skin (45). In addition, PPARα expression in epidermis is reduced in similar hapten-induced murine AD models, and topical activation of PPARα exhibits a substantial therapeutic effect on murine AD, by restoring permeability barrier function and by dampening allergic inflammation (46, 47).

Awareness of physiological properties of PPARα in the skin and the association of decreased PPARα expression with AD suggests that PPARα might be one of the macromolecules that participates in “intrinsic” cross talk. In fact, reduction of PPARα expression by transfection with siRNA against *PPARα* not only up-regulates expression of the Regulated-upon-activation, normal-t cell-expressed-and-presumably-secreted cytokine (RANTES) and the Thymus- and activation-regulated chemokine (TARC) in cultured keratinocytes, but also down-regulates expression of transglutaminase 1 and LOR (48), further suggesting that PPARα modulates functions associated both with inflammation and with permeability barrier homeostasis in skin.

## Discussion

A vicious cycle involving permeability barrier dysfunction and allergic inflammation is one of the basic mechanisms leading to the pathogenesis of AD. In this article, in addition to discussing the concept of the so-called “outside-to-inside, and back-to-outside” paradigm, we offer an idea that links permeability dysfunction and allergic inflammation in the pathogenesis of AD. In the “outside-to-inside and back-to-outside” model, keratinocyte functions are modified secondarily by external stimuli, such as SC pH, or certain inflammatory factors. On the other hand, in our “intrinsic” model, primary functional changes relevant to both permeability barrier and inflammation can occur in keratinocytes by alteration of even a single molecule. Secondary alteration of such a molecule may also contribute to augment the vicious cycle between permeability barrier dysfunction and allergic inflammation. This concept demonstrates the importance of keratinocytes as a key player in the pathogenesis of AD, although it is unclear whether this concept applies equally to extrinsic and intrinsic AD.

Keratinocytes are well known to have functions related to both permeability barrier dysfunction and inflammation, meaning that keratinocytes likely are involved in both of these processes in the pathogenesis of AD. The concept “intrinsic” highlights molecules in keratinocytes which are simultaneously involved in both permeability barrier function and inflammation, and such molecules could be candidates as therapeutic targets in AD, as in the case of PPARα activators in the murine AD model. Interestingly, the Janus kinase inhibitor, JTE-052, now the main ingredient in an ointment called Delgocitinib, was originally reported to induce filaggrin expression, and this ointment has been deployed as topical therapy for AD (49, 50), suggesting that targeting keratinocyte functions is one potential strategy for treating AD.

Knowledge that changes in one molecule in keratinocytes can lead to both permeability barrier abrogation and inflammation has been already reported, as in the case of KRT1 mutations (27). Moreover, Akiyama et al. have described an autoinflammatory keratinization disease paradigm (51–53), and this disease concept seems almost identical to inflammatory aspects in our “intrinsic” mechanism described here. Therefore, seeking the pathomechanisms of autoinflammatory keratinization diseases could help to clarify the mechanism underlining our “intrinsic” paradigm, and AD might be recognized as an autoinflammatory keratinization disease in the future.

## Author contributions

YH wrote the first draft of the manuscript. PE revised the manuscript. All authors contributed to manuscript revision, read, and approved the submitted version.
